# Accelerating Policy Decisions to Adopt *Haemophilus influenzae* Type b Vaccine: A Global, Multivariable Analysis

**DOI:** 10.1371/journal.pmed.1000249

**Published:** 2010-03-16

**Authors:** Jessica C. Shearer, Meghan L. Stack, Marcie R. Richmond, Allyson P. Bear, Rana A. Hajjeh, David M. Bishai

**Affiliations:** 1 Johns Hopkins Bloomberg School of Public Health, Baltimore, Maryland, United States of America; 2 Hib Initiative, Baltimore, Maryland, United States of America; Emory University, United States of America

## Abstract

**Background:**

Adoption of new and underutilized vaccines by national immunization programs is an essential step towards reducing child mortality. Policy decisions to adopt new vaccines in high mortality countries often lag behind decisions in high-income countries. Using the case of *Haemophilus influenzae* type b (Hib) vaccine, this paper endeavors to explain these delays through the analysis of country-level economic, epidemiological, programmatic and policy-related factors, as well as the role of the Global Alliance for Vaccines and Immunisation (GAVI Alliance).

**Methods and Findings:**

Data for 147 countries from 1990 to 2007 were analyzed in accelerated failure time models to identify factors that are associated with the time to decision to adopt Hib vaccine. In multivariable models that control for Gross National Income, region, and burden of Hib disease, the receipt of GAVI support speeded the time to decision by a factor of 0.37 (95% CI 0.18–0.76), or 63%. The presence of two or more neighboring country adopters accelerated decisions to adopt by a factor of 0.50 (95% CI 0.33–0.75). For each 1% increase in vaccine price, decisions to adopt are delayed by a factor of 1.02 (95% CI 1.00–1.04). Global recommendations and local studies were not associated with time to decision.

**Conclusions:**

This study substantiates previous findings related to vaccine price and presents new evidence to suggest that GAVI eligibility is associated with accelerated decisions to adopt Hib vaccine. The influence of neighboring country decisions was also highly significant, suggesting that approaches to support the adoption of new vaccines should consider supply- and demand-side factors.

*Please see later in the article for the Editors' Summary*

## Abstract 2

Jessica Shearer and colleagues analyze data from 147 countries to identify factors that influence the time taken to introduce routine vaccination against *Haemophilus influenzae* type b (Hib).

## Editors' Summary

**Background:**

Every year, immunization averts more than 2 million deaths by preparing people's immune systems to recognize and attack disease-causing organisms (pathogens) rapidly and effectively. Although the immune system is designed to protect the human body against infections, the first time a person is exposed to a pathogen (usually during early childhood) their immune system can take some time to respond. As a result, they can become seriously ill or even die. However, the immune system “learns” from the experience and when the pathogen is encountered again, the immune system swings into action much more quickly. Immunization or vaccination is a safe way to make individuals resistant to infectious diseases. It works by exposing them to weakened or dead pathogens or to pathogen molecules (antigens) that the immune system recognizes as foreign. Widespread, routine immunization of children is, therefore, an essential component of national and global strategies to reduce childhood illnesses and deaths.

**Why Was This Study Done?:**

Although many factors affect the uptake of immunization (in particular, vaccine prices), national policy decisions to adopt new vaccines are an essential step toward improving coverage. Unfortunately, these decisions are often delayed in developing countries. Thus, although many industrialized countries have routinely immunized their children with the highly effective *Haemophilus influenza* type b (Hib) conjugate vaccine since it became available in the early 1990s, only 13 low-income countries were using the vaccine in 2004. Hib bacteria, which cause pneumonia (lung infection) and meningitis (brain inflammation), kill about 370,000 unvaccinated young children every year. In this study, the researchers try to explain delays in the adoption of routine Hib vaccination in developing countries by analyzing the associations between Hib vaccination and factors such as national economic status, local Hib burden, and eligibility for support from the Global Alliance for Vaccines and Immunisation (GAVI Alliance; a public–private partnership that offers financial, technical, and health systems support for the introduction of national immunization programs to developing countries that meet certain eligibility criteria).

**What Did the Researchers Do and Find?:**

The researchers used a statistical approach called accelerated failure time analysis to analyze data collected in 147 countries between 1990 and 2007 on vaccine costs, Hib disease incidence, GAVI eligibility, and other factors that could influence decision-makers' perceptions of the costs and benefits of Hib vaccination. After allowing for gross national income, region, and burden of Hib disease, the researchers identified several factors that influenced the time between the availability of a Hib conjugate vaccine in a country and a decision being made to introduce routine Hib vaccination. The receipt of GAVI support speeded the decision to adopt vaccination by 63%, for example, and sharing borders with two or more countries that had adopted the vaccine speeded the decision by 50%. By contrast, for each 1% increase in vaccine costs, the time to decision to adopt vaccination was delayed by 2%. The 1998 and 2006 World Health Organization recommendations on routine Hib vaccination and the existence of local studies on Hib disease had no influence on the time to decision.

**What Do These Findings Mean?:**

These findings confirm previous studies that showed that increases in the price of Hib vaccine increase the time to adoption. In addition, they suggest that GAVI eligibility accelerates decisions to adopt this vaccine and show that the decisions made by neighboring countries are important, which suggests that policy diffusion may occur. Thus, in the case of adoption of the Hib vaccine, both supply-side and demand-side factors seem to be important. Its is relevant to note that during writing of the article, JCS, MLS, MRR, APB, and RAH were employed by the Hib Initiative, which was funded by the GAVI Alliance. The findings do not necessarily represent the views, policies or decisions of the Hib Initiative or the GAVI Alliance. Importantly, these findings are explanatory, not predictive, so they cannot be applied directly to new vaccines to improve their rate of adoption. Nevertheless, these findings highlight the potential importance of setting up formal and informal networks to facilitate policy diffusion and suggest that long-term price and supply certainty might be factors that could help to accelerate national decisions to adopt new and/or underutilized vaccines and other public-health technologies.

**Additional Information:**

Please access these Web sites via the online version of this summary at http://dx.doi.org/10.1371/journal.pmed.1000249.

- The World Health Organization provides information on immunization and on Haemophilus influenza type b (in several languages)
- The GAVI Alliance Web site describes the work of this public–private partnership and provides details of developing countries eligible for Hib vaccination support
- The Hib Initiative aims to reduce the risk of childhood death and disability through sustained use of Hib vaccine
- MedlinePlus provides links to further resources on immunization and information on the Haemophilus influenzae type b vaccine (in English and Spanish)

## Introduction

Routine immunization is an essential component of strategies to reduce childhood mortality, but its impact is limited by delays in national decision-making, national and subnational implementation, and low coverage [1]. While not sufficient, national policy decisions to adopt vaccines are a necessary condition for their routine implementation and widespread use.

*Haemophilus influenzae* type b (Hib) conjugate vaccine prevents morbidity and mortality resulting from Hib disease, which is estimated to cause 370,000 deaths each year in unvaccinated children under five years of age [2]. Where routinely used, Hib vaccines have reduced morbidity and mortality from Hib disease [3]–[7]. Decisions to adopt Hib vaccine in low- and middle-income countries have lagged behind high-income countries. Its routine use was confined to 13 low-income countries in 2004; by the end of 2008, 66 low-income countries had made a decision to adopt Hib vaccine [8]. In order to explain the increased frequency of decisions to adopt Hib vaccine since 2004 and to inform efforts related to other public health interventions, it is useful to understand what influenced Hib vaccine adoption decisions.

Recent literature has utilized qualitative and quantitative methods to study decisions to adopt hepatitis B virus (HBV), Hib, and typhoid vaccines (see Table 1 for a list and description of studies). Findings from these studies suggest that national vaccine decision-making is driven by vaccine price [9],[10], existence of local studies or disease burden data [9]–[14], immunization system strength [9],[13]–[15], donor financing for vaccines [13]–[15], and national economic and political characteristics [15].

**Table 1:** Previous findings on factors that influence national vaccine decision-making.

Author	Important Variables	Methods	Vaccine
**Widdus (1999) ** **[31]**	• Local burden of disease and vaccine effectiveness studies• Cost-effectiveness studies• Cost reduction and knowledge sharing platforms for poorer countries• International recommendations• Private sector use• Social/behavioral research to understand local barriers to acceptance• Advocacy for economic benefit of vaccine• Emerging market technology licensing• Coordinating approval process of vaccines in all countries• Donor financing	Conceptual framework of suggested actions to accelerate vaccine introduction in low-income countries	New vaccines
**Wenger et al. (2000) ** **[11]**	• Published burden of disease and effectiveness studies• Local experience with vaccine• Support from pediatrician association• Price• Public interest (political will)• Surveillance data	Case study of four early adopting, nonindustrialized countries	Hib
**Miller and Flanders (2000) ** **[9]**	• DTP3 coverage• GDP per capita• Vaccine cost (price per dose)• Treatment cost per unit• Years of life lost• Years of life lost• Treatment cost prevented• Years of life saved	Univariate logistic analysis	HBV
**Gauri and Khaleghian (2002) ** **[15]**	• Democracy score• GDP per capita• DTP3 coverage• Presence of UNICEF financing• PAHO revolving fund membership• Institutional quality score	Longitudinal multivariable analysis	HBV
**DeRoeck et al. (2005) ** **[12]**	• Burden of disease studies• Cost-effectiveness studies• Vaccine price• Vaccine safety and immunogenicity• Feasibility of local production• WHO recommendation• Ease of inclusion in current EPI	Qualitative interviews with country-level policy makers and immunization professionals	Cholera, typhoid fever, shigellosis
**Munira and Fritzen (2007) ** **[13]**	• External pressures and advocacy• Burden of disease studies• Scientific support on benefit of vaccine• Feasibility of adoption into current EPI• Local vaccine pilot studies	Case studies of two early adopting countries (Taiwan, Thailand)	HBV
**Rossi et al. (2007)** **[14]**	• Geographical region• DTP3 coverage• Previous introduction of HBV• GNI per capita	Cross-sectional, descriptive comparison	Hib
**Danovaro-Holliday et al. (2008)** **[10]**	• Political will• Burden of disease and impact studies• “Experience exchange” between countries• PAHO Revolving Fund membership	Descriptive analysis and case studies from the Americas	Hib

Studies were identified by searching for keyword combinations of “Hib,” “new vaccine,” or “immunization” and “policy,” “adoption,” or “government factors” in PubMed, SCOPUS, PAIS International, EconLit, and WorldCat. Additional articles were identified by reviewing references of retrieved studies.

The recent increase in decisions to adopt Hib vaccine enables a longitudinal analysis of previously identified factors, as well as the addition of variables to test new hypotheses. This paper describes the results of a multivariable analysis of the factors that may have shortened the length of time between vaccine availability and a country's decision to adopt Hib vaccine. As new vaccines and other public health technologies become available, a better understanding of the factors that are associated with accelerated adoption of appropriate technologies could speed health improvements for populations around the world.

## Methods

The study outcome is a policy decision of a country to universally adopt Hib vaccine. Compared with programmatic implementation, which can vary subnationally, policy decisions correspond to a discrete time point at the national level. For Global Alliance for Vaccines and Immunisation (GAVI)-eligible countries, decision year is defined as the year that a country applied to GAVI for new vaccine financial support. GAVI Alliance, a global public-private partnership, was created in 2000 to improve access to underutilized vaccines, including Hib vaccine (see Box 1). For non-GAVI–eligible countries, we use the year that Hib vaccine first appeared in the country's immunization schedule, as dates of policy decisions were not available [16]. Because a decision can occur only once, countries are censored from the model the year following a decision.

**Box 1. GAVI Background**

GAVI offers financial, technical, and health systems support to national immunization programs in the world's 72 poorest countries (GNI per capita less than $1,000; 2003 USD). These 72 countries are all considered GAVI-eligible, but to apply for support, countries must also have a functional interagency coordinating committee and DTP3 coverage >50%.

**Available support**

Eligible countries can apply to GAVI for:

- Cofinancing support for new and underused vaccines (HBV, Hib, yellow fever, pneumococcal, rotavirus, measles)
- Immunization services support (flexible funding to improve immunization performance)
- Injection safety support
- Health systems strengthening (flexible funding to target health systems constraints)
- Civil society organization support

In addition to offering these forms of “hard” support, GAVI also engages in global advocacy, liaises with national decision-makers and stakeholders, and funds vaccine-specific initiatives such as the Hib Initiative.

**Funding received**

As of July 2009, 71 countries had submitted successful applications for various forms of GAVI support, including cofinancing for new and underused vaccines (*n* = 49); health systems strengthening (*n* = 28); immunization services support (*n* = 61); and injection safety support (*n* = 66).

Countries entered the model when a Hib-containing conjugate vaccine was first available for use in their country. This corresponded to the year of Hib conjugate licensure (1990) for most high- and middle-income countries, and the year of WHO prequalification (1998) for countries that procure vaccines through UNICEF. Of 193 countries reporting data to WHO and World Bank, 19 were excluded from the model because of missing explanatory data >10%.

Explanatory variables were proposed on the basis of existing literature. A core model included four explanatory variables. Additional variables were chosen to further describe contextual factors, the costs and benefits of introducing Hib vaccine, and factors that modify decision-makers' perception of these costs and benefits (Table S1). Fourteen variables were included in the final model according to these categories and the constraints of the statistical model (Table 2). These variables and our hypotheses of their effects are described below.

**Table 2:** Summary statistics of included independent variables.

Variables	Type	Median (Lower; Upper Quartile) or N (%)^a^
***Context***		
Natural log population (1,000s)	Continuous	15.67 (14.16; 17.96)
Region: High-income OECD	Binary	137 (7.95)
East Asia and Pacific	Binary	209 (12.12)
Europe and Central Asia	Binary	282 (16.36)
Latin America and Caribbean	Binary	291 (16.88)
Middle East and North Africa	Binary	149 (8.64)
Other high-income	Binary	153 (8.87)
South Asia	Binary	60 (3.48)
Sub-Saharan Africa	Binary	443 (25.7)
DTP3 coverage (%)	Continuous	88 (75; 96)
Democracy score	Continuous	5 (−4; 8)
***Costs and benefits***		
Natural log price per dose (US$)	Continuous	1.26 (−1.17; 1.51)
Natural log GNI (US$)	Continuous	22.80 (21.43; 24.58)
Natural log cost per bed-day (Int$)	Continuous	4.02 (3.39; 4.54)
Incidence of Hib disease (per 100,000 child-years)	Continuous	820 (450; 1562)
Availability of Hib in combination with DTP and/or HBV	Binary	304 (17.60)
***Modifying factors***		
Local incidence/disease burden studies	Continuous	0 (0; 0)
Neighboring adopters: None	Binary	1,130 (65.55)
One neighboring country adopter	Binary	331 (19.2)
Two or more neighboring country adopters	Binary	263 (15.26)
WHO position paper: None	Binary	688 (39.91)
First WHO position	Binary	890 (51.62)
Second WHO position	Binary	146 (8.47)
GAVI eligibility	Binary	432 (25.1)
Cofinancing uncertainty	Binary	148 (8.58)

a N = country-years.

Variables to describe the underlying country context are geopolitical region, coverage of three doses of diphtheria, tetanus, and pertussis vaccine (DTP3) [17], and democracy score [18]. The model controls for population size [17]. DTP3 coverage is used as a proxy for immunization system strength, and we expect a positive association between DTP3 and accelerated decision-making. Level of democracy was captured using an existing continuous scale (democracy score) that quantifies type of governance, ranging from fully institutionalized autocracy (−10) to fully institutionalized democracy (+10) [18]. We expect that decision-making was accelerated in more democratic states [15].

Costs and benefits of Hib vaccine introduction were measured by vaccine price per dose, cost per bed-day (International $; Int$) [19], Hib disease incidence [2], and the availability of Hib-containing combination vaccines (DTP-HBV-Hib and DTP-Hib). This latter construct was represented by a dummy variable for years 2002–2007. The model controls for gross national income (Atlas US$; abbreviated as GNI) [17]. Vaccine price per dose was collected from public sources [20]–[24], published economic analyses [25]–[27], and personal communication with country governments. For GAVI-eligible countries, we used the subsidized price that they would pay after GAVI's cofinancing contribution. For non-GAVI–eligible countries where data were unavailable, the median price for that country's income strata was used. To reflect historical changes in price for countries paying a private market price (i.e., not UNICEF or GAVI pricing), we applied the trajectory of the historical price trend paid by the US government [23]. We expect an association between decreases in vaccine price and accelerated decision-making [9],[10]. Cost per bed-day [19] was converted to year-specific costs using GDP deflators and purchasing power parity data available from the World Bank World Development Indicator database [17]. Estimates of country-level Hib disease incidence were obtained from the Hib and Pneumococcal Global Burden of Disease Study [2]. While high disease incidence should encourage policy action, low national income in high-burden countries suggests that the perceived costs of vaccination may have outweighed the perceived benefits in these settings. We expect that Hib vaccine will be more attractive to decision-makers in combination with other antigens and will be associated with accelerated decision-making.

To measure factors that could modify a decision-maker's perception of the costs and benefits of Hib vaccine and its position on the policy agenda, we include variables to describe the existence of local Hib studies, decisions of neighboring countries, WHO recommendations [3],[28], GAVI eligibility [29], and cofinancing uncertainty. The number of high-quality published local studies is measured by a continuous variable and based on a bibliography of Hib disease burden studies [30]. We hypothesize that local studies would have increased general awareness of Hib disease amongst decision-makers, thus accelerating time to decision [10]–[13],[31]. Number of neighboring adopters was determined by mapping vaccine introduction for each year. A country was defined as having “*N*” neighboring adopters if “*N*” countries sharing an international border had already adopted Hib vaccine. Island countries were considered to share a border with geographically proximate islands and mainland countries. Decision-makers have indicated through interviews that neighboring countries' actions influence their own decisions [10],[13],[32] and we expect to find the same.

The WHO has published two recommendations regarding Hib vaccine. The 1998 position paper states that countries should consider Hib burden before introducing the vaccine [28]; a 2006 modification recommends the inclusion of Hib vaccine in all routine immunization programs, regardless of national burden [3]. Both position papers are represented by separate dummy variables for their respective years, and we anticipate that they accelerated time to decision.

Since 2000, countries with GNI per capita less than $1,000 (2003 US$) have been eligible to apply for support from GAVI (Box 1) [29]. GAVI eligibility is a time-varying binary variable. Whether or not a country applies for or receives support, they are coded as one (GAVI-eligible) if they meet GAVI eligibility criteria in years 2000–2007 and zero (non-GAVI–eligible) if they do not meet eligibility criteria; all countries are coded as zero in years before 2000. Because the final model includes a variable for price per dose, the GAVI variable represents GAVI-funded advocacy and technical support external to actual financial disbursements for new vaccines. We hypothesize that being GAVI-eligible modified decision-makers' awareness of Hib vaccination and elevated its position on the policy agenda. The cofinancing uncertainty variable accounts for a period of time (2004–2006) when GAVI was in the process of revising its financing policy. This dummy variable flags observations for GAVI-eligible countries from 2004 to 2006 to explore the relationship between price uncertainty and the lower incidence of decisions to introduce during those years.

Survival analysis was used to model how time-varying factors affect the duration of time between vaccine availability and a country's policy decision to adopt. Survival functions and their differences are described by Kaplan-Meier curves and by log-rank tests, respectively. For multivariable analyses, we used an accelerated failure time (AFT) model in preference to a semiparametric proportional hazard model, as AFT models are more robust to the presence of unobservable confounders for each country. However, they require parametric assumptions regarding both the distribution of the time to event and the distribution of the frailty parameter [33]. Our study explored the robustness of the results of a basic set of variables to the Weibull [34], log-normal and log-logistic distributions and to both gamma and Gaussian frailty assumptions. Choice of model did not significantly alter the regression coefficients or results of the log-likelihood test (Table S2); however, a Weibull distribution with Gaussian frailty was chosen as the best fit. Variables were added to a core model using manual forward stepwise selection that considered theoretical hypotheses. The final model of 14 independent variables was selected based on the results of likelihood ratio and Akaike information criteria [35]. Sensitivity analysis measured possible overadjustment for income by rerunning the model without GNI. Analysis was performed with Stata statistical software version 10.0 (College Station, TX).

## Results

**Figure 1:**
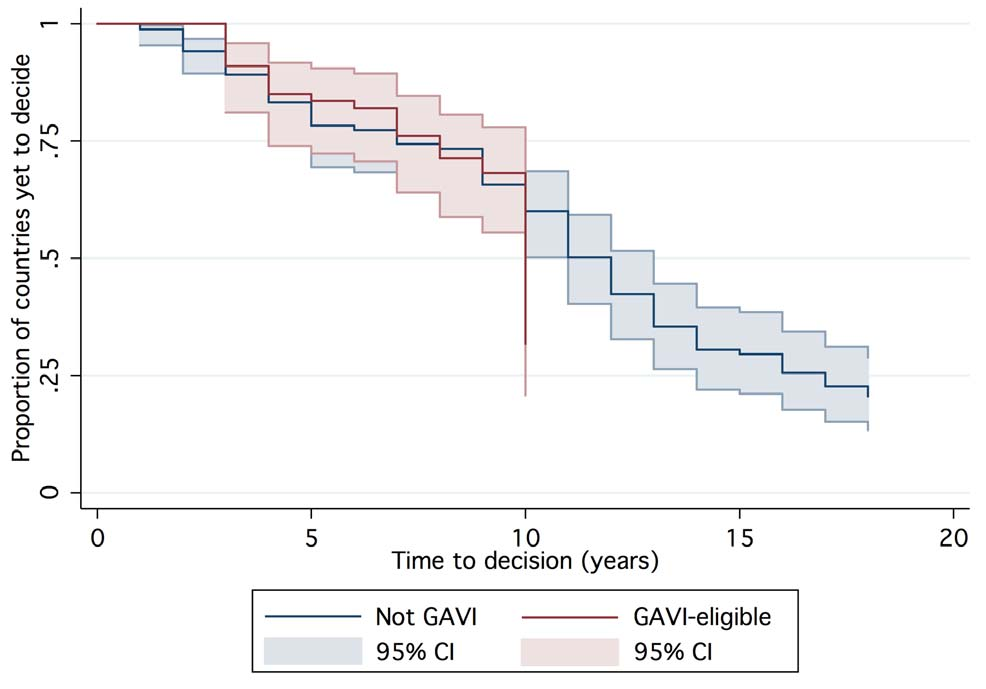
Time to decision by GAVI and non-GAVI countries. Time = 0 represents first availability (licensure) of Hib vaccine (1998 for UNICEF procurers; 1990 for others).

**Figure 2:**
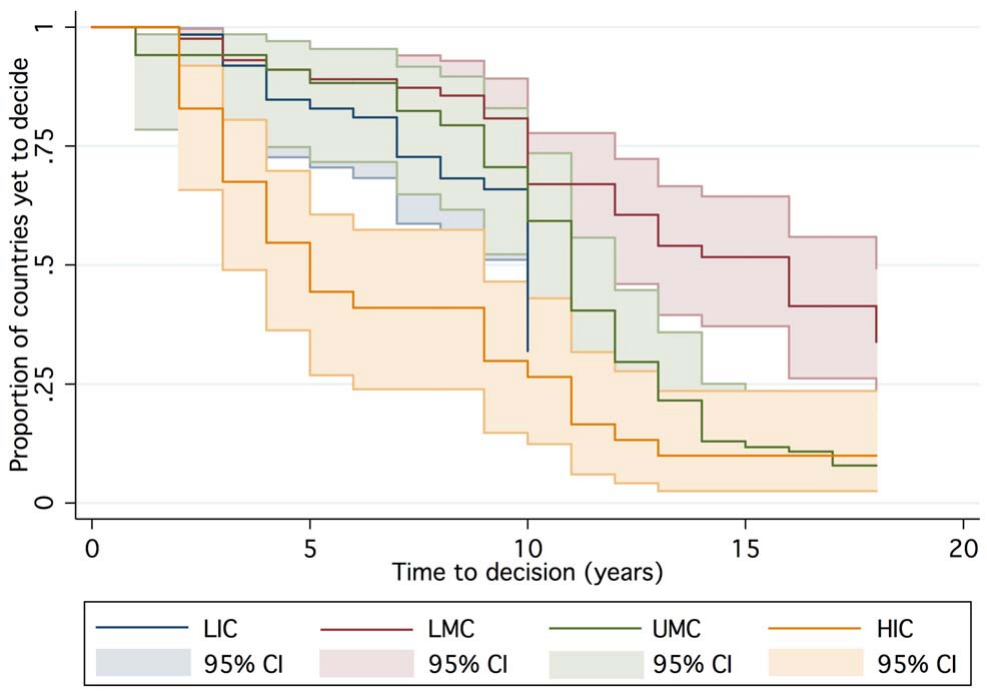
Time to decision by World Bank income group. See [17].

**Figure 3:**
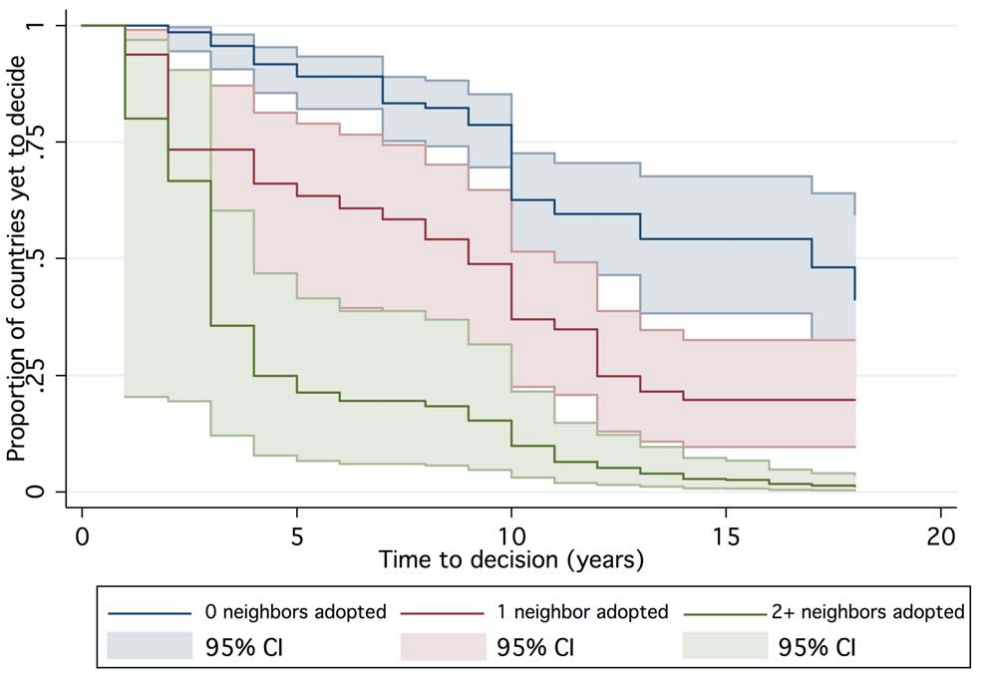
Time to decision by neighboring country adopters.

One hundred and forty-seven countries were amenable to analysis in the final AFT model, accounting for 1,383 country-years of observation between 1990 and 2007. By 2007, 39 countries had not yet made a policy decision to adopt Hib vaccine and were right censored, which was taken into consideration by the likelihood model [36]. The Kaplan-Meier survival curves depict the time from licensure (availability) of vaccine to decision. While the GAVI and non-GAVI curves follow similar trajectories for the first nine years, the significant acceleration observed for GAVI-eligible countries in year 10 (2007; see Figure 1) results in a statistically significant difference between the curves overall (log rank test *p* = 0.002). In Figure 2, GAVI eligibility appears to correct for differences between income levels (*p*<0.001 comparing low-income countries (LIC) with lower-middle income countries (LMC); *p* = 0.04 comparing LIC with upper-middle income countries (UMC)). Figure 3 demonstrates the effect of neighboring countries' decisions: bordering two or more adopters accelerates decision-making (log rank test *p*<0.001). This effect held for both GAVI-eligible and non-GAVI–eligible countries.

In the multivariable AFT model, the effects of individual variables on time to decision were measured while controlling for all other modeled variables (see Table 3). The exponentiated coefficients in Table 3 describe the factor by which the expected time to decision is multiplied for each unit increase in the independent variable, all other variables held constant. Coefficients less than one indicate accelerated time to decision.

**Table 3:** Results from accelerated failure time model.

Variables	Proportionate Change in Time to Decision	95% CI	*p*-Value
***Context***			
Natural log population (1,000s)	1.14	[0.89–1.46]	0.285
Region (compared to high-income OECD)			
East Asia and Pacific	2.47*	[1.02–5.95]	0.044
Europe and Central Asia	3.33**	[1.67–6.67]	0.001
Latin America and Caribbean	1.10	[0.57–2.12]	0.775
Middle East and North Africa	1.56	[0.70–3.44]	0.277
Other high-income	1.19	[0.63–2.23]	0.599
South Asia	2.58	[0.80–8.27]	0.112
Sub-Saharan Africa	1.40	[0.56–3.37]	0.448
DTP3 coverage (%)	0.99	[0.98–1.00]	0.059
Democracy score	0.97*	[0.94–1.00]	0.041
***Costs and benefits***			
Natural log price per dose (US$)	1.02*	[1.00–1.04]	0.015
Natural log GNI (US$)	0.92	[0.72–1.18]	0.530
Natural log cost per bed-day (Int$)	1.00	[0.81–1.22]	0.972
Incidence of Hib disease (per 100,000 child-years)	1.02	[0.97–1.07]	0.434
Availability of Hib in combination with DTP and/or HBV	1.13	[0.63–1.99]	0.686
***Modifying factors***			
Local incidence/disease burden studies	0.92	[0.83–1.02]	0.123
Neighboring adopters (compared to none)			
One neighboring country adopter	0.67*	[0.48–0.94]	0.021
Two or more neighboring country adopters	0.50**	[0.33–0.75]	0.001
WHO position paper (compared to none)			
First WHO position	0.94	[0.57–1.53]	0.787
Second WHO position	0.59	[0.26–1.32]	0.198
GAVI eligibility	0.37**	[0.18–0.75]	0.007
Cofinancing uncertainty	2.26**	[1.23–4.15]	0.009
Constant	28.69	[0.82–994.53]	0.064

*p<0.05, **p<0.01, Weibull distribution, Gaussian frailty. *n* = 1,383.

In the multivariable AFT model, non-OECD (Organisation for Economic Co-operation and Development) European and Central Asian countries' time to decision was lengthened by a factor of more than three (95% CI 1.67–6.66) as compared to the high-income OECD reference group. Countries in the East Asia and Pacific region experienced a delay of over two times (95% CI 1.02–5.95). DTP3 coverage was not statistically significantly associated with time to decision, but each increase in degree of democracy – for example, the difference between Argentina in 1991 and in 1999 [18]—shortened time to decision by a factors of 0.97 (95% CI 0.94–0.999).

Each 1% increase in price per dose was associated with a 1.02 times longer time to decision (95% CI 1.00–1.04), controlling for national income. Neither cost per bed-day, incidence of Hib disease, nor availability of Hib in a combination vaccine were statistically significant.

The existence of local studies was not statistically significantly related to a change in time to decision. However, decision-making was accelerated by decisions to adopt Hib vaccine in neighboring countries. Bordering one neighboring decider shortened the time to adoption by a factor of 0.67 (95% CI 0.48–0.94) while two or more neighboring deciders shortened the time to adoption by 0.50 (95% CI 0.33–0.75). Neither of the WHO position statements was statistically significantly associated with time to decision.

Being GAVI-eligible shortened the time to decision by a factor of 0.37 (95% CI 0.18–0.76) holding vaccine price and all other covariates constant. Exposure to the period of uncertain financing (2004–2006) mediated GAVI's effect, shortening time to decision by 0.83 (the GAVI coefficient multiplied by the cofinancing uncertainty coefficient). The final model was minimally sensitive to GNI; removing GNI reduced the GAVI coefficient by three percentage points.

## Discussion

Consistent with our hypotheses, adoption time was associated with the costs and benefits of Hib immunization, and by factors that might influence decision-makers' perceptions of these costs and benefits. Notably, being GAVI-eligible was associated with a significant accelerating effect on time to decision. The principal mechanisms through which GAVI could hasten adoption decisions, independent of financing, likely include advocacy efforts, interpersonal contact with national decision-makers, and technical support. The high degree of correlation of these efforts across GAVI countries does not permit efforts to disentangle them. The creation of the GAVI-funded Hib Initiative in 2005 may explain the surge of decisions in GAVI-eligible countries in 2007 (see Figure 1, year 10), but too few country-years exist to confirm or refute this hypothesis.

The presence of two or more neighboring adopters accelerated time to decision by a factor of 0.50, suggesting a process of policy diffusion as has been indicated through qualitative research [10],[13],[32]. In this model, WHO recommendations did not accelerate time to decision. Rather than minimizing the saliency of such recommendations, the aggregate of the findings suggests that their influence could be strengthened through dissemination via regional networks.

Previous research has described the influence of local evidence on decision-making [9]–[14], a finding that was not substantiated here. However, we were not able to model the results of local studies, only their existence. Knowing that studies exist is not equivalent to knowing the implications of their findings or their dissemination to decision-makers. At the same time, the influence of local studies is not necessarily proportional to their quality or validity. Limited data and poor model fit prevented attempts to model the actual findings of local incidence studies, and the local incidence estimates from the Global Burden of Disease study were nonsignificant, perhaps due to regional heteroskedasticity generated by their modeling process.

Divergent from our hypotheses and earlier research [9], our model found no association between immunization system strength, as measured by DTP3 coverage, and time to decision to introduce the Hib vaccine. Considering the potential for collinearity between DTP3 coverage and national income, it is conceivable that the surge of decisions in low-income countries masks any potential effect of DTP3.

### Strengths and Weaknesses of the Study

To our knowledge, this study is the first to use longitudinal, multivariable methods to describe Hib vaccine policy decisions, and to incorporate the influence of GAVI and neighboring countries. Our model is explanatory rather than predictive, thus its findings cannot be directly applied to newer vaccines. Nor could our model include all possible covariates or countries; 19 countries were excluded due to missing explanatory data. Based on Student t-tests, these countries were earlier deciders (*p* = 0.008), had smaller populations (*p*<0.001), and were less democratic (*p*<0.001) than the included countries. Findings from this analysis may not be generalizable to the excluded countries. Covariates were excluded because of model constraints, missing data, and difficulties in operationalizing certain theoretical constructs, including those related to political and historical influence, institutions, actors, and advocacy.

Finally, the choice of entry time into our model artificially underestimates the true delay in access for most low- and middle-income countries. We consider these countries to enter at the time of WHO licensure (1998), which was eight years after the vaccine's US licensure. This eight-year delay is related to issues of supply and cost. The choice of entry time, while reflective of available policy options in developing countries, may bias results towards faster adoption in GAVI-eligible countries.

### Implications

Although limited to explaining decision patterns of Hib vaccine, these findings may be of interest to other public health programs with similar goals. For example, they highlight the potential of formal and informal networks to facilitate policy diffusion, including regional organizations (e.g., PAHO), regional forums and meetings (e.g., WHO Expanded Programme on Immunization [EPI] manager meetings), and new-vaccine application workshops. Recent global interest in facilitating South-South learning and cooperation should be encouraged and explored further.

Vaccine price remains a significant barrier for many middle-income countries, but the expansion of bulk purchasing models similar to the PAHO revolving fund or the Gulf Cooperation Council group purchasing model offers promise of secure and affordable supply [37]. However, findings from our model substantiate historical evidence that suggests that price alone will not drive decision-making. At GAVI's inception, countries could obtain Hib vaccine for free, yet the rate of adoption decisions increased only after a cofinancing requirement was enacted (year 10 in Figure 1). In fact, the effect of price uncertainty during GAVI's period of policy transition (2004–2006) was larger than the effect of the vaccine price itself. These findings suggest that long-term price and supply certainty should be prioritized for other new vaccines.

This study demonstrates the importance of both supply- and demand-side approaches in accelerating decisions to adopt cost-effective, underutilized public health technologies. In the cases of human papillomavirus, pneumococcal, and rotavirus vaccines, GAVI's new Accelerated Vaccine Introduction initiative may wish to consider additional factors unique to these newer vaccines.

### Further Research

Further research should attempt to operationalize additional theoretical constructs of policy decision-making and measure them prospectively. Measuring the level and intensity of various GAVI activities could better elucidate mechanisms through which GAVI might accelerate decision-making. Measurement of domestic decision-making processes, including the influence of national immunization technical advisory committees, would be an important contribution.

Future research should explore the effect of neighboring countries' research evidence, including disease burden and vaccine impact studies. As policy decisions are implemented, future analyses should measure time to implementation. In addition, evaluation of programmatic outcomes, including coverage and lives saved, will inform discussions related to timely access and programmatic effectiveness.

Progress toward meeting national and global health and development targets depends on appropriate national policies. We hope that these findings inform efforts to support evidence-based policy decisions to adopt proven, cost-effective public health interventions.

## Supporting Information

Table S1 Variables considered.
(0.06 MB DOC)

Table S2 Results of accelerated failure time (AFT) models with various distributions and frailty assumptions.
(0.06 MB DOC)

## Abbreviations

AFT: accelerated failure time
DTP3: diphtheria-tetanus-pertussis vaccine
EPI: Expanded Programme on Immunization
GAVI [Alliance]: Global Alliance for Vaccines and Immunisation
GNI: gross national income
Hib: *Haemophilus influenzae* type b
HBV: hepatitis B virus
HIC: high-income country
Int$: international dollar
LIC: low-income country
LMC: lower-middle income country
OECD: Organisation for Economic Co-operation and Development
PAHO: Pan-American Health Organization
UMC: upper-middle income country
WHO: World Health Organization
