# Supplementary material for: Accelerating Policy Decisions to Adopt Haemophilus influenzae Type b Vaccine: A Global, Multivariable Analysis
Source: PLoS Med. 2010 Mar 16;7(3):e1000249. doi: 10.1371/journal.pmed.1000249 (PMC2838745; doi:10.1371/journal.pmed.1000249)
Supplement: Table S1 — Variables considered. (0.06 MB DOC) [file pmed.1000249.s001.doc]

Table S1: Variables considered

| **Variables for which data was collected** | **Type of variable** | **Timing** | **Reason for inclusion/exclusion** |
| --- | --- | --- | --- |
| ***Context*** |  |  |  |
| Total population | Continuous | Annual | Included |
| Geopolitical region | Categorical | Constant | Included |
| DTP3 coverage | Continuous | Annual | Included |
| Democracy score | Continuous | Annual | Included |
| Gini coefficient | Continuous | Annual | Missing Data |
| Decentralization | Continuous | Annual | Missing Data |
| Life expectancy at birth | Continuous | Annual | Repeated/Collinear |
| Surviving infants | Continuous | Annual | Repeated/Collinear |
| Birth rate per 1000 population | Continuous | Annual | Repeated/Collinear |
| Birth cohort (live births) | Continuous | Annual | Repeated/Collinear |
| Under 5 mortality rate | Continuous | Annual | Repeated/Collinear |
| Institutional quality | Continuous | Annual | Repeated/Collinear |
| PAHO | Binary | Constant | Repeated/Collinear |
| ***Costs and Benefits*** |  |  |  |
| Vaccine price per dose | Continuous | Annual | Included |
| Gross national income | Continuous | Annual | Included |
| Cost per bed-day in tertiary care facility | Continuous | Annual | Included |
| Hib incidence | Continuous | Annual | Included |
| % of total health expenditure that is private | Continuous | Annual | Did not converge in final model |
| Total spending on routine immunization, US$ | Continuous | Annual | Missing Data |
| Immunization spending per DTP3 immunized child | Continuous | Annual | Missing Data |
| Immunization spending per capita | Continuous | Annual | Missing Data |
| % routine immunizations funded by the Government | Continuous | Annual | Missing Data |
| Routine immunizations as % of total health expenditures | Continuous | Annual | Missing Data |
| Availability of combination vaccine | Binary | Annual | Included |
| ***Modifying Factors*** |  |  |  |
| Cumulative number of disease burden studies that exist each year in country | Continuous | Annual | Included |
| Cumulative indicator of neighboring country introduction | Categorical | Annual | Included |
| WHO recommendation on routine Hib vaccine use | Categorical | Annual | Included |
| GAVI eligibility (=0 for all countries prior to 2000, =1 for any country-year that a country was eligible for GAVI assistance) | Binary | Annual | Included |
| Co-financing uncertainty | Binary | Annual | Included |
| Cost-effectiveness study done in country | Binary | Annual | Did not converge in final model |
| Paediatric advocacy | Binary | Annual | Missing Data |
| Existence of safety/immunogenicity study | Binary | Annual | Missing Data |
| Hib Rapid Assessment Tool (RAT) performed in country | Binary | Annual | Repeated/Collinear |
