# Supplementary material for: Accelerating Policy Decisions to Adopt Haemophilus influenzae Type b Vaccine: A Global, Multivariable Analysis
Source: PLoS Med. 2010 Mar 16;7(3):e1000249. doi: 10.1371/journal.pmed.1000249 (PMC2838745; doi:10.1371/journal.pmed.1000249)
Supplement: Table S2 — Results of accelerated failure time (AFT) models with various distributions and frailty assumptions. (0.06 MB DOC) [file pmed.1000249.s002.doc]

**Table S2: Results of AFT models with various distributions and frailty assumptions**

|  | **Coefficients and standard errors by distribution and frailty assumptions** | | | | |
| --- | --- | --- | --- | --- | --- |
| **Variables** | **Weibull/ Gaussian** | **Weibull/ gamma** | **Log-normal/ Gaussian** | **Log-normal /gamma** | **Log-logistic/ gamma*** |
| Natural log population | 1.557** | 1.557** | 1.508** | 1.508** | 1.470** |
|  | [0.140] | [0.140] | [0.162] | [0.162] | [0.149] |
| Natural log GNI | 0.713** | 0.713** | 0.748** | 0.748** | 0.739** |
|  | [0.0536] | [0.0535] | [0.0645] | [0.0645] | [0.0642] |
| GAVI eligibility | 0.386** | 0.388** | 0.429** | 0.429** | 0.426** |
|  | [0.0838] | [0.0844] | [0.119] | [0.119] | [0.113] |
| One neighboring country adopter | 0.616** | 0.617** | 0.529** | 0.529** | 0.619** |
|  | [0.110] | [0.109] | [0.108] | [0.108] | [0.115] |
| Two or more neighboring country adopters | 0.352** | 0.353** | 0.243** | 0.243** | 0.340** |
|  | [0.0737] | [0.0732] | [0.0706] | [0.0706] | [0.0897] |
| Constant | 62.00** | 62.07** | 25.45** | 25.44** | 49.64** |
|  | [59.59] | [59.82] | [28.34] | [28.34] | [56.64] |
| Log Likelihood | -125 | -125 | -128.1 | -128.1 | -130.6 |
| ** p<0.01, * p<0.05 |  |  |  |  |  |

*Convergence was not achieved with the log-logistic distribution with Gaussian frailty assumption.
